# Evaluation of Neuromuscular Diseases and Complaints by Quantitative Muscle MRI

**DOI:** 10.3390/jcm13071958

**Published:** 2024-03-28

**Authors:** Lara Schlaffke, Robert Rehmann, Anne-Katrin Güttsches, Matthias Vorgerd, Christine H. Meyer-Frießem, Hubert R. Dinse, Elena Enax-Krumova, Martijn Froeling, Johannes Forsting

**Affiliations:** 1 Department of Neurology, BG-University Hospital Bergmannsheil, Ruhr University Bochum, 44789 Bochum, Germany; 2 Department of Neurology, Klinikum Dortmund, University Witten-Herdecke, 44137 Dortmund, Germany; 3 Heimer Institute for Muscle Research, BG-University Hospital Bergmannsheil, 44789 Bochum, Germany; 4 Department of Anaesthesiology, Intensive Care and Pain Management, St. Marien Hospital, 44534 Lünen, Germany; 5 Department of Anaesthesiology, Intensive Care Medicine and Pain Management, BG-University Hospital Bergmannsheil, Faculty of Medicine, Ruhr University Bochum, 44789 Bochum, Germany; 6 Department of Radiology, University Medical Center Utrecht, 3584 CX Utrecht, The Netherlands

**Keywords:** quantitative muscle MRI, muscle diffusion tensor imaging, skeletal muscle, neuromuscular disorders, inclusion body myositis, limb-girdle muscular dystrophy, Pompe disease, post-COVID-19-condition

## Abstract

**Background:** Quantitative muscle MRI (qMRI) is a promising tool for evaluating and monitoring neuromuscular disorders (NMD). However, the application of different imaging protocols and processing pipelines restricts comparison between patient cohorts and disorders. In this qMRI study, we aim to compare dystrophic (limb-girdle muscular dystrophy), inflammatory (inclusion body myositis), and metabolic myopathy (Pompe disease) as well as patients with post-COVID-19 conditions suffering from myalgia to healthy controls. **Methods**: Ten subjects of each group underwent a 3T lower extremity muscle MRI, including a multi-echo, gradient-echo, Dixon-based sequence, a multi-echo, spin-echo (MESE) T2 mapping sequence, and a spin-echo EPI diffusion-weighted sequence. Furthermore, the following clinical assessments were performed: Quick Motor Function Measure, patient questionnaires for daily life activities, and 6-min walking distance. **Results**: Different involvement patterns of conspicuous qMRI parameters for different NMDs were observed. qMRI metrics correlated significantly with clinical assessments. **Conclusions**: qMRI metrics are suitable for evaluating patients with NMD since they show differences in muscular involvement in different NMDs and correlate with clinical assessments. Still, standardisation of acquisition and processing is needed for broad clinical use.

## 1. Introduction

Neuromuscular disorders (NMD) include several hundreds of different diseases caused by malfunctions in peripheral nerves, the neuromuscular junction, and muscle [1]. NMDs primarily affecting the skeletal muscle can have different aetiologies, ranging from autoimmune inflammatory to hereditary metabolic or dystrophic. Idiopathic inflammatory myopathies can often be fulminant, with inclusion body myositis (IBM) being the most common in older people [1]. The most often hereditary muscular dystrophies are Duchenne and Becker in children and facioscapulohumeral dystrophy (FSHD) in adults [2]. The most common metabolic myopathies are glycogenosis, Pompe, and McArdle disease [3]. Other possible causes of NMD are environmental chemical exposure, poisoning, and viral and bacterial infections [4]. In the wake of the COVID-19-pandemic, virus-associated neuromuscular complaints have gained attention [5]. Persistent myalgia and premature muscular fatigue affect at least 25% of post-COVID-19-condition patients (PCC), defined as persistent symptoms after confirmed SARS-CoV-2 infection that occurs within three months from the onset of COVID-19 and lasts for at least two months without an alternative explanation [6,7]. At the same time, the role of persisting subclinical muscle inflammation or postinfectious structural abnormalities in contributing to clinical symptoms in PCC remains unclear and needs further investigation [8].

In specific diseases like spinal muscle atrophy (SMA), new therapeutic principles, such as splice modulation or gene therapy, have recently led to a breakthrough in treatment with different pharmacological mechanisms and medications available [9]. In contrast to these advances, several hundred NMDs remain untreatable, leading to progressive muscle wasting and functional impairment. Outside of the technical complexities inherent in developing personalised therapies, the execution of clinical trials for these diseases is further complicated by their low prevalence and characteristically slow progression [10]. Additionally, limitations of classic clinical assessments, such as subjectivity of ratings and rater dependency, have been discussed [11]. Therefore, different instrumental and laboratory techniques were recently proposed as suitable biomarkers that provide information about disease progression and evaluate and ideally predict treatment response [12].

Quantitative muscle MRI (qMRI) has emerged as a non-invasive and objective tool for assessing and monitoring NMD [13,14]. Through their ability to accurately derive individual muscle fat fractions (FF), Dixon-based sequences help identify typical patterns of muscular involvement [15,16,17]. They also can detect subtle disease progressions, even before clinical symptoms and assessments deteriorate [18,19]. Water T2 relaxation time measurements allow the evaluation of inflammation and myoedema, illustrating fluid retention in affected tissues [20,21]. Muscle diffusion tensor imaging (mDTI) can reflect muscle micro- and macrostructure by tracing the movement of water molecules, which are restricted by inherent cellular boundaries [22,23]. mDTI metrics detected structural abnormalities such as fibre size alterations in different NMDs, such as Becker and Duchenne muscular dystrophy [24,25]. mDTI tractography can illustrate muscle fibre tracts reflecting the muscular macrostructure [26,27]. However, comparing qMRI studies between different sites and diseases is complicated due to the lack of standardised imaging protocols and data analysis [10,28].

Therefore, in this qMRI study—using a previously established protocol including mDTI—we aim to compare various patient cohorts and provide recommendations for the clinical application of qMRI in the daily routine [29]. These cohorts include a dystrophic NMD (limb-girdle muscular dystrophy), an inflammatory NMD (inclusion body myositis), a metabolic myopathy (Pompe disease), the post-COVID-19 condition with neuromuscular complaints, as well as healthy controls.

## 2. Materials and Methods

### 2.1. Study Population

In total, 40 individuals with genetically confirmed limb-girdle muscular dystrophy (LGMD) R1, probable or definite inclusion body myositis (IBM) according to the ENMC criteria, late-onset Pompe disease (LOPD), and post-COVID-19-condition (PCC) were included in this study (10 per group). The PCC all reported muscular complaints, such as myalgia and premature muscular fatigue. Furthermore, ten healthy volunteers were assessed. The exclusion criteria for healthy volunteers included a medical history of NMD and lower extremity injuries within the 12 months before study enrolment. Informed consent was obtained from all participants. Table 1 provides an overview of clinical data for the different study groups.

### 2.2. Clinical Assessments

Muscle strength was evaluated by experienced clinicians using the Quick Motor Function Measure (QMFM) [30]. Daily life activities were assessed in myopathies using the ACTIVLIM and the Neuromuscular Symptom Score (NSS) questionnaires [18,19]. For mobility assessment, an experienced medical technical assistant obtained the 6-minute walking distance (6-MWD) in ambulatory individuals.

### 2.3. MRI Acquisition and Processing

Participants were positioned supine in a Philips 3.0T Achieva MR system (Philips, Best, The Netherlands), with their feet entering first. Sandbags were placed around their feet to minimise movement, and cushions supported their knees. Both lower extremities were scanned using a 16CH Torso XL coil (Philips, Best, The Netherlands). The thigh region, from hip to knee, was covered by two fields of view (FOV) measuring 480 × 276 × 150 mm^3^ each along the *z*-axis (stacks), designed to reduce shimming artefacts associated with larger FOVs. These stacks overlapped by 30 mm to facilitate precise merging with the distal edge at the crotch area. A single FOV of the exact dimensions was used for the leg region, with its proximal edge 60 mm below the tibial plateau, aligned perpendicular to the tibial bone.

The scanning protocol included a Dixon-based sequence (voxel size 1.5 × 1.5 × 6.0 mm^3^; TR/TEs 210/2.6, 3.36, 4.12, 4.88 ms; flip angle 8°, SENSE: 2), a multi-echo spin-echo (MESE) sequence for quantitative water mapping with 17 echoes and Cartesian k-space sampling (voxel size 3.0 × 3.0 × 6.0 mm^3^; TR/TE 4598/17 × ∆7.6 ms; flip angle 90/180°, SENSE: 2), and a diffusion-weighted spin-echo EPI (voxel size 3.0 × 3.0 × 6.0 mm^3^; TR/TE 5000/57 ms; SPAIR/SPIR fat suppression; SENSE: 1.9; 42 gradient directions with eight b-values ranging from 0–600) [29]. A noise scan was also conducted using the same parameters as the DWI but without RF power and gradients (only acquisition channels open).

Data preprocessing followed previously described methods using qMRITools 3.16.0, running under Wolfram Mathematica 14.0 (https://github.com/mfroeling/QMRITools) [29,31]. Briefly, the Dixon data were processed using iterative decomposition of water and fat with echo asymmetry and least-squares estimation, accounting for a single T2* decay, resulting in separated water and fat maps [32]. The water maps thus obtained were used for manual segmentation. T2-mapping data were analysed using an EPG fitting approach, considering different T2 relaxation times for water and fat components [33]. The diffusion data were denoised using a principal component analysis [34]. Each lower extremity underwent separate registration to correct for subject motion and eddy currents. Tensors were computed accounting for intravoxel incoherent motion using an iterative weighted linear least squares algorithm [35,36].

Eight thigh muscles (vastus lateralis, vastus medialis, rectus femoris, semimembranosus, semitendinosus, biceps femoris—long and short head, sartorius, and gracilis) and seven leg muscles (extensor digitorum, gastrocnemius lateralis and medialis, peroneal group, soleus, tibialis anterior, and tibialis posterior) were segmented within the acquired FOVs using an automated tool and further refined by an experienced rater in both lower extremities [37].

The segmentations were aligned with T2 and DTI data to correct for motion between sequences and eddy current distortions, using sequential rigid and b-spline transformations [38]. Average values of proton density FF and water T2 relaxation time were calculated over all slices. SNR was determined as previously described [39].

For the diffusion data analysis, whole muscle tractography was conducted for each muscle within muscle segmentations to evaluate muscle architecture. Tracking parameters included a maximum angle of 15°, fibre length range of 20 to 500 mm, FA range of 0.05–0.65, and MD range of 0.25–2.5 10^−3^ mm^2^/s [40]. DTI parameters such as fractional anisotropy (FA), mean diffusivity (MD), axial diffusivity (λ_1_), and radial diffusivity (RD) were obtained using tract-based sampling.

### 2.4. Statistical Analysis

Descriptive statistics were used to describe the study population features and qMRI parameters for each muscle separately. MRI outcome parameters were compared to clinical outcome measures by calculating compound scores for all thigh and leg muscles, considering each muscle’s segmentation mask volume [13]. Pearson correlation coefficients were calculated for metric values to explore relationships between MRI parameters and clinical outcomes, and Spearman correlation coefficients were used for rank values.

Differences in qMRI metrics between study groups were assessed in a multivariate general linear model with patient/control, body side, and muscle as fixed factors using BMI and gender as covariates. The same multivariate general linear model was used to analyse diffusion metrics, excluding muscles with an FF higher than 60% and an SNR lower than ten after denoising, suggesting low diffusion data quality [15,25]. Subsequently, post hoc tests were performed using Bonferroni correction. All statistical analyses were conducted using IBM SPSS V28 (IBM Corp, Armonk, NY, USA), with a significance level of *p* < 0.05.

## 3. Results

**Figure 1:**
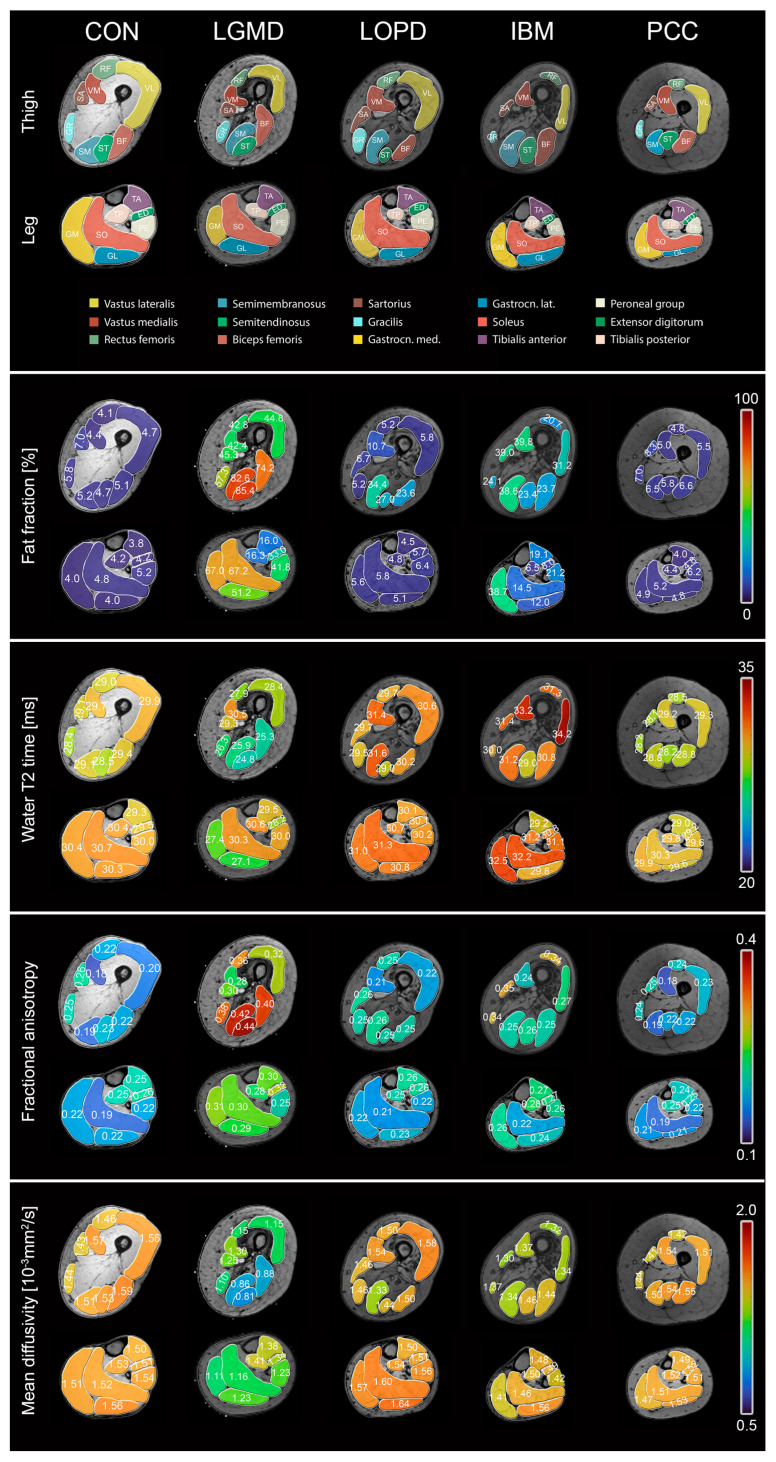
Overview of mean qMRI parameters for different study groups and muscles. A representative cross-section of the thigh and leg is displayed for each parameter map and study group. Fat fraction: Different patterns of fat replacement can be observed. The highest mean fat fractions (FF) were found in LGMDR1 patients, primarily affecting the hamstrings (semimembranosus, semitendinosus, biceps femoris) in the thigh and the triceps surae (gastrocnemius medialis, gastrocnemius lateralis, soleus) in the leg. LOPD patients showed an initial involvement of the hamstrings, particularly the semimembranosus muscle. In IBM patients, the highest fat fraction was seen in the vastus medialis and sartorius muscle in the thigh and the gastrocnemius medialis in the leg, while the tibialis posterior was spared. In PCC, no significant differences can be observed compared to CON. Water T2 time: In LGMDR1 patients, the lowest water T2 relaxation time values were observed, particularly in the highly fat-replaced hamstrings and gracilis muscles. In LOPD, water T2 relaxation times were elevated compared to controls in both thigh and leg muscles. In IBM, the highest water T2 relaxation time values were noted, especially in the vastus medialis and vastus lateralis in the thigh and the gastrocnemius medialis and soleus in the leg, potentially reflecting active muscle damage. PCC showed a slight tendency towards lower T2 relaxation times compared to CON. Fractional anisotropy: The highest fractional anisotropy (FA) values were seen in LGMDR1 in all muscles, especially the fat-replaced hamstrings. Similar tendencies were observed for all thigh muscles in IBM and hamstrings in LOPD. In PCC, FA values are slightly elevated compared to controls. Mean diffusivity: The lowest mean diffusivity (MD) values were observed in LGMDR1 and IBM. LOPD and PCC showed similar values compared to healthy controls except for a low MD in the semimembranosus of Pompe patients.

**Figure 2:**
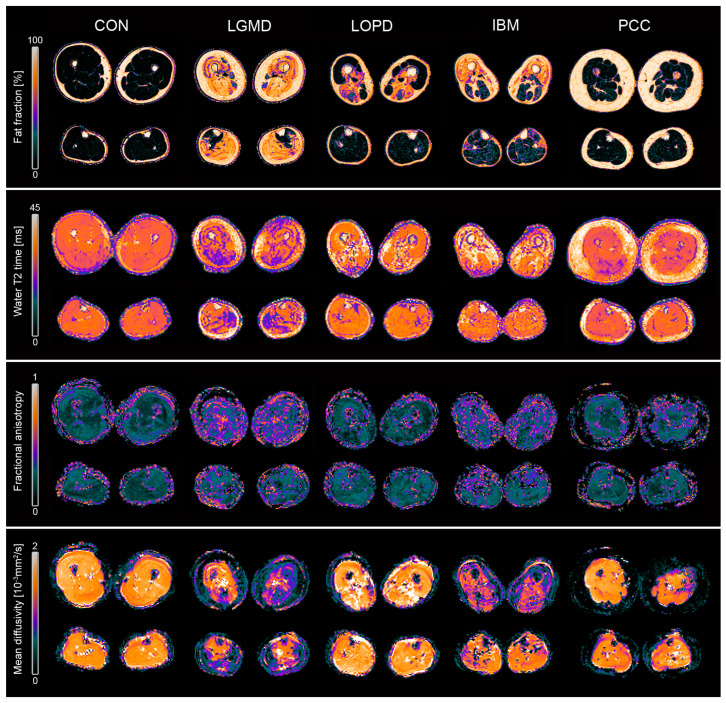
Representative examples of different qMRI parameter maps for healthy controls (CON), limb-girdle muscular dystrophy R1 (LGMD), late-onset Pompe disease (LOPD), inclusion body myositis (IBM), and post-COVID-19-condition (PCC). Fat fraction: Differences in fat replacement are displayed, showing high FF in all muscles of LGMDR1, sparing the tibialis anterior and tibialis posterior. LOPD patients showed fat replacement exclusively in the posterior thigh. In IBM, the anterior part of the thigh is predominantly affected. Water T2 time: The lowest water T2 relaxation time values were observed in LGMDR1 patients, particularly in the posterior part of the thigh. In LOPD, water T2 relaxation times were elevated compared to controls in both thigh and leg muscles. In IBM, high water T2 values are depicted in the anterior part of the thigh despite high-fat replacement, reflecting active muscle damage. Fractional anisotropy: LGMDR1 and IBM show higher fractional anisotropy (FA) values compared to controls. Mean diffusivity: LGMDR1 and IBM displayed lower mean diffusivity values than controls.

**Figure 3:**
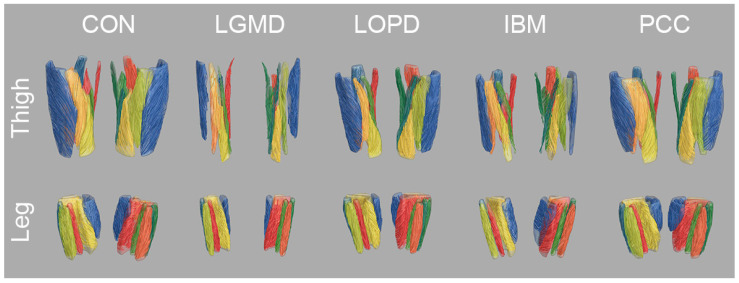
Overview of tractography results in representative participants for healthy controls (CON), limb-girdle muscular dystrophy R1 (LGMD), late-onset Pompe disease (LOPD), inclusion body myositis (IBM), and post-COVID-19-condition (PCC). The reconstructed fibre tracts reflect the macrostructure of the underlying muscle tissue. Despite high fat replacement in LGMD and IBM, mDTI tractography can reconstruct fibre tracts corresponding to the anatomical basics. However, disrupted fibres can be observed in highly affected muscles, especially in the thigh muscles of LGMD and IBM.

Clinical assessments were successfully performed on all participants except for 6-MWD in non-ambulatory individuals (n = 7). Mean values and standard deviations of clinical outcome measures are shown in Table 1. Scans were successfully conducted in all participants except for two LOPD patients who did not tolerate the full scanning protocol and only underwent leg muscle MRI. Figure 1 (top rows) shows all segmented muscles and Figure 2 gives an overview of parameter maps of qMRI metrics in representative participants. Figure 3 shows tractography results in representative participants.

### 3.1. Fat Fraction

Different patterns of fat replacement were found for LGMDR1, IBM, and LOPD, as illustrated in Figure 1 and Table S1. Participants with PCC exhibited FF comparable to those of healthy controls. The highest FF were observed in LGMDR1 patients, primarily affecting the hamstrings (semimembranosus, semitendinosus, biceps femoris) in the thigh and the triceps surae (gastrocnemius medialis, gastrocnemius lateralis, soleus) in the leg. In IBM patients, the highest fat fraction was seen in the vastus medialis and sartorius muscle in the thigh and the gastrocnemius medialis in the leg. LOPD patients showed a beginning involvement of the hamstrings, especially the semimembranosus muscle. GLM showed significant differences in FF between study groups (*p* < 0.001). Post hoc tests showed that FF were significantly higher than CON in LGMDR1 (mean difference: +46.6 ± 1.4%, *p* < 0.001), IBM (mean difference: +17.0 ± 1.4%, *p* < 0.001), and LOPD (mean difference: +6.3 ± 1.4%, *p* < 0.001). FF in PCC and CON did not show significant differences (*p* = 0.913). The FF compound scores for all thigh and leg muscles are displayed for each group in Table 2.

### 3.2. Water T2 Relaxation Time

CON showed mean water T2 values between 28.4 and 29.9 ms in the thigh and between 29.3 and 30.7 ms in the leg (see Figure 1, Table S1). The compound score for water T2 relaxation time was comparable between CON and PCC (see Table 2). The highest water T2 relaxation time values were observed in IBM, especially in the vastus medialis and vastus lateralis in the thigh and gastrocnemius medialis and soleus in the leg. In contrast, the lowest water T2 relaxation time values were found in LGMDR1, especially in the hamstrings and gracilis muscles. GLM revealed significant differences in T2 between the study groups (*p* < 0.001). Post hoc tests showed that water T2 relaxation time was higher in IBM (mean difference: +1.4 ± 0.2 ms, *p* < 0.001) and LOPD (mean difference: +0.9 ± 0.2 ms, *p* < 0.001) in comparison to CON, while LGMD (−1.5 ± 0.2 ms, *p* < 0.001) and PCC (−0.8 ± 0.2 ms, *p* < 0.001) displayed lower water T2 values than CON.

**Table 2:** Compound scores of qMRI metrics fat fraction (FF), water T2 relaxation time (T2), fractional anisotropy (FA), and mean diffusivity (MD) for leg and thigh muscles of different study groups.

		LGMDR1	IBM	LOPD	PCC	CON
		Mean ± SD	Mean ± SD	Mean ± SD	Mean ± SD	Mean ± SD
Thigh	FF [%]	56.2 ± 23.7	26.6 ± 12.3	22.2 ± 7.3	5.8 ± 1.2	4.8 ± 0.4
	T2 [ms]	27.6 ± 2.9	31.8 ± 1.4	30.3 ± 1.0	28.9 ± 0.9	29.4 ± 0.6
	FA	0.35 ± 0.07	0.26 ± 0.02	0.24 ± 0.05	0.22 ± 0.02	0.21 ± 0.01
	MD [10^−3^ m/s^2^]	1.08 ± 0.25	1.40 ± 0.11	1.50 ± 0.08	1.51 ± 0.04	1.54 ± 0.03
Leg	FF [%]	50.0 ± 18.0	16.8 ± 15.0	5.7 ± 3.4	5.0 ± 1.0	4.4 ± 0.7
	T2 [ms]	29.5 ± 1.8	31.5 ± 0.6	31.0 ± 0.8	29.9 ± 0.9	30.3 ± 0.7
	FA	0.29 ± 0.07	0.24 ± 0.03	0.23 ± 0.04	0.21 ± 0.01	0.21 ± 0.01
	MD [10^−3^ m/s^2^]	1.22 ± 0.20	1.48 ± 0.13	1.58 ± 0.09	1.50 ± 0.04	1.52 ± 0.04

LGMDR1—limb-girdle muscular dystrophy R1; IBM—inclusion body myositis, LOPD—late-onset Pompe disease; PCC—post-COVID-19 condition; CON—healthy controls; FF—fat fraction; T2—water T2 relaxation time; FA—fractional anisotropy; MD—mean diffusivity.

### 3.3. Diffusion Metrics

CON displayed compound scores for mean FA of 0.21 in both thigh and leg, while compound scores for mean MD varied from 1.52 to 1.54. PCC showed slightly higher FA and lower MD values than healthy controls (see Table 2). The highest FA and lowest MD values were found in LGMDR1, affecting the hamstrings especially (see Table 2, Figure 1). Similar tendencies were observed for the thigh muscles in IBM and hamstrings in LOPD. GLM showed significant differences in FA and MD between study groups (*p* < 0.001). Post hoc tests showed that FA in LGMDR1 (mean difference: +0.043 ± 0.004, *p* < 0.001), IBM (mean difference: +0.043 ± 0.003, *p* < 0.001), LOPD (mean difference: +0.011 ± 0.003, *p* = 0.006), and PCC (mean difference: +0.012 ± 0.004, *p* = 0.011) were significantly higher than CON. Post hoc tests for MD showed significantly lower values in LGMDR1 (mean difference: −0.152 ± 0.013, *p* < 0.001) and IBM (mean difference: −0.066 ± 0.010, *p* < 0.001) compared to CON. No significant differences in MD were observed between CON and LOPD (*p* = 1.0) and CON and PCC (*p* = 0.258).

### 3.4. Correlation with Clinical Findings

Correlations between clinical assessments and qMRI values are displayed in Table 3 and Table S2. Significant correlations between 6-MWD and FF of thigh muscles were observed in LGMDR1 (r = −0.932, *p* < 0.01) and LOPD (r = −0.798, *p* < 0.05) but not for IBM (r = −0.353, *p* = 0.352). Similar correlations were found for QMFM and FF of thigh muscles (LGMDR1: r = −0.875, *p* < 0.01; IBM: r = −0.511, *p* = 0.132; LOPD: r = −0.847, *p* < 0.05). Significant negative correlations of T2 in thigh muscles with 6-MWD and QMFM were found for IBM (see Table 3). No significant correlations were found for CON. For PCC, a significant negative correlation of MD in leg muscles with 6-MWD was found (r = −0.884, *p* < 0.01).

## 4. Discussion

In this cross-sectional descriptive study, we explored and compared different NMD using qMRI, demonstrating its feasibility and utility in assessing NMD. qMRI metrics can capture disease-specific patterns of muscle involvement and correlate with clinical outcome measures across degenerative, inflammatory, and metabolic myopathies. These findings underscore the potential of qMRI metrics as a biomarker for assessing muscle tissue functionality, differentiating muscular involvement patterns, and monitoring NMD.

The most established qMRI method involves the evaluation of intramuscular fat content through Dixon-based sequences [28]. This technique detects nuanced disease progression, often preceding clinical symptom deterioration [19]. Furthermore, it has proved valuable in recognising common patterns of muscle involvement. Our study enables us to comprehend previously described involvement patterns, such as the predominant involvement of the hamstrings in the thigh and the soleus and gastrocnemius medialis in the leg in LGMDR1 [41,42]. In IBM, the vastus medialis and sartorius muscle in the thigh and the gastrocnemius medialis in the leg were most affected, consistent with prior semiquantitative and quantitative MRI studies [43,44,45]. In LOPD, hamstring muscles were predominantly affected, with complete sparing of leg muscles. Notably, the FF compound score of thigh muscles exhibited significant correlations with 6-MWD and QMFM, underlining the clinical relevance of the evaluation of fat replacement. Previous studies in LOPD have shown that Dixon-derived FF is more sensitive to longitudinal changes than clinical assessments [46]. The ability to detect subtle changes in muscle fat content using qMRI holds significant promise for early diagnosis and precise monitoring of disease progression and treatment efficacy. However, fat replacement is irreversible and is the common final pathway in most NMD. Water T2 relaxation time and diffusion metrics have been hypothesised to detect early pathophysiological changes in NMD before the irreversible process of fat replacement [47,48].

Water T2 relaxation time is expected to increase with oedema, inflammation, or general muscle damage. However, water T2 values also decrease with increasing FF, which can be explained by fibrotic modelling in advanced NMD since water T2 values of fibrotic tissue are as low as 10 ms [49,50]. This phenomenon could explain the notably reduced T2 relaxation times in LGMDR1, primarily driven by exceedingly low water T2 values in thigh muscles with high-fat replacement. To avoid the influence of fat replacement, water T2 values have been assessed as early detection in low-fat-replaced muscles [15,51]. However, the underlying pathophysiological mechanism leading to reduced water T2 values in PCC remains elusive and has not been observed in more significant cohorts of PCC patients [52]. In metabolic diseases like LOPD, elevated water T2 values have been linked to accelerated disease progression. However, the precise pathophysiological mechanisms of elevated T2 values in LOPD in our study are still unclear [53]. In contrast, despite ongoing fat replacement, the elevated water T2 values in IBM align with the underlying combined degenerative and inflammatory pathophysiology characteristic of IBM [54]. The correlations of water T2 values in thigh muscles of IBM and LOPD with clinical outcome measures underline the clinical impact of those observations. Moreover, a recent study by Wang et al. was able to associate elevated water T2 values in idiopathic inflammatory myopathies with the degree of myofiber size variation, inflammatory cell infiltration, and amount of connective tissues [20]. Therefore, water T2 values could serve as a non-invasive biomarker of disease activity, especially in inflammatory myopathies. However, water T2 relaxation is unspecific since it is sensitive to various processes, reflecting different pathophysiological mechanisms [20,51].

Other promising candidates in the assessment of NMD are diffusion metrics, reflecting the movement of water molecules within muscle tissue [22]. These metrics can detect structural abnormalities in non-fat-replaced muscles and have shown sensitivity in fat-replaced muscles using adequate fat suppression methods [15,47,55]. However, highly effective fat suppression can lead to images dominated by noise. The random nature of this noise erroneously would cause the diffusion to appear very anisotropic [56]. This effect is particularly pronounced in tissues with high fat content, primarily due to the low water content of adipose tissue. Therefore, these considerations could impact the descriptive statistics in Figure 1, which show high anisotropic diffusion in high-fat-replaced muscles. To minimise the influence of these effects in the statistical analysis, diffusion data with low SNR values were excluded. An increase in FA and a decrease in MD, as observed in LGMDR1 and IBM, were interpreted as a sign of fibre atrophy, which is also supported by simulation experiments [55,57]. Interestingly, the FA increase with decreasing muscle fibre size can precede MD changes, which could account for the observations in LOPD. However, different pathophysiological mechanisms, such as glycogen accumulation or autophagic deposits, have been discussed to explain diffusion changes in LOPD [47,58]. In our study, the tendency towards higher MD values was mainly driven by leg muscles, which also displayed higher water T2 values than CON. The water T2 elevation has been formerly interpreted as a sign of disease activity in LOPD [53]. MD values showed a strong, significant negative correlation with QMFM in thigh muscles but not with 6-MWD, potentially reflecting pronounced axial weakness in LOPD better captured by QMFM. These findings need further investigation since changes in diffusion metrics in low-fat-replaced thigh muscles of LOPD have been described before [47]. However, studies cannot be compared directly due to different acquisition protocols and processing pipelines. In PCC, the significant strong negative correlation between MD and 6-MWD could hint at microstructural changes which go along with worse clinical performance. However, no significant differences between PCC and CON were observed. The absence of structural anomalies in PCC may be due to the relatively small cohort size, which is insufficiently powered to detect the subtle differences from CON.

Considering these findings, qMRI emerges as a helpful marker in the assessment of NMD. Different qMRI patterns in the investigated patient population may be caused by different pathophysiological mechanisms, which may impact each other. Hence, the various modalities should always be considered together to obtain a comprehensive perspective. Especially, FF measures are essential to account for the influence of fat replacement on water T2 relaxation time and diffusion metrics. However, adaptations of imaging protocols for shorter examination times may be warranted to enhance clinical acceptance and patient compliance, such as Dixon-based sequences for the follow-up of high-fat-replaced musculature. Disease-specific considerations, such as sparing of leg muscles in LOPD, should also be factored in. However, the heterogeneity of muscle involvement in the different study groups suggests that muscle-wise segmentation is necessary for detailed assessment and early disease-specific change detection, although costly and time-consuming. Future applications of qMRI could include disease monitoring, prognosis assessment, clinical trials, and treatment selections since the first applications have shown promising results [19,59,60]. As mentioned, direct comparison of qMRI study results is often restricted due to different and complex acquisition and processing pipelines. Using standardised imaging and data analysis in our study ensures direct comparability of different diseases. In the future, consensus criteria for imaging, at least for evaluation of muscular fat content using Dixon-based sequences and T2 mapping, should be developed to guarantee a direct comparison between centres and diseases.

There are some limitations in this study. The qMRI techniques used in this study are still non-standard MR acquisition, requiring sophisticated, time-consuming postprocessing. Further simplification and automation are essential for widespread clinical use. Moreover, due to the low prevalence of NMD, group sizes were relatively low, warranting future studies to pool patients from different centres to ensure larger group sizes.

## 5. Conclusions

In this study, we showed that qMRI depicts differences in cohorts of patients with different NMD. Furthermore, qMRI reveals different muscular involvement patterns and correlates with clinical assessments, endorsing that qMRI is a suitable non-invasive method for evaluating patients with NMD. Potential future uses of qMRI may encompass disease monitoring, prognosis assessment, participation in clinical trials, and selection of treatment modalities. However, standardised and simplified imaging protocols and data analysis are needed to ensure the broad deployment of qMRI techniques and comparability between different centres.

## Figures and Tables

**Table 1:** Clinical data of patients with limb-girdle muscular dystrophy (LGMD) R1, inclusion body myositis (IBM), late-onset Pompe disease (LOPD), post-COVID-19-condition (PCC), and healthy controls (CON).

Group	Sex (m/f)	Age (Years)	BMI	Disease Duration (Years)	QMFM	NSS	ACTIVLIM	6-MWD (Meter)
LGMDR1	5/5	39.6 ± 12.1	23.0 ± 5.0	22.2 ± 7.3	28 ± 16	30 ± 7	31 ± 4	316 ± 78
IBM	5/5	69.3 ± 6.1	27.1 ± 4.2	8.8 ± 3.9	39 ± 11	30 ± 8	28 ± 10	387 ± 142
LOPD	5/5	47.2 ± 18.9	22.8 ± 3.3	12.8 ± 6.4	49 ± 14	22 ± 2	35 ± 7	461 ± 71
PCC	5/5	50.3 ± 11.0	28.7 ± 3.7	1.2 ± 0.4	64 ± 0	-	-	458 ± 84
CON	5/5	46.1 ± 12.8	23.4 ± 2.1	-	64 ± 0	-	-	569 ± 45

QMFM—Quick Motor Function Measure; NSS—Neuromuscular Symptom Score; ACTIVLIM—Active Limitation; 6-MWD—6-minute walking distance.

**Table 3:** Overview of Pearson and Spearman correlation coefficients for qMRI parameters fat fraction (FF), water T2 relaxation time (T2), fractional anisotropy (FA), and mean diffusivity (MD), and clinical outcome measures 6-minute walking distance (6-MWD), and Quick Motor Function Measure (QMFM).

			LGMDR1	IBM	LOPD	PCC	CON
Thigh	6-MWD	FF	−0.932 **	−0.353	−0.798 *	−0.491	0.264
		T2	0.328	−0.795 *	−0.701	0.682	−0.099
		FA	−0.808 *	−0.523	−0.527	0.095	0.079
		MD	0.889 **	0.481	−0.177	−0.475	0.403
	QMFM	FF	−0.875 **	−0.511	−0.847 *	-	-
		T2	0.012	−0.717 *	−0.991 **	-	-
		FA	−0.766 **	−0.553	0.324	-	-
		MD	0.918 **	0.553	−0.775 *	-	-
Leg	6-MWD	FF	−0.200	−0.371	−0.330	−0.578	0.236
		T2	0.713	0.035	0.328	0.652	−0.238
		FA	−0.443	−0.722 *	−0.248	0.219	−0.010
		MD	−0.159	0.690	−0.319	−0.884 **	0.182
	QMFM	FF	−0.322	−0.225	−0.814 *	-	-
		T2	−0.322	−0.498	−0.407	-	-
		FA	−0.310	−0.213	−0.299	-	-
		MD	0.286	0.097	−0.695	-	-

LGMDR1—limb-girdle muscular dystrophy R1; IBM—inclusion body myositis; LOPD—late-onset Pompe disease; PCC—post-COVID-19 condition; CON—healthy controls. * *p* < 0.05, ** *p* < 0.01.

## Data Availability

The data that support the findings of this study are available from the corresponding author, but restrictions apply to the availability of these data, which were used under license for the current study and so are not publicly available.

## Supplementary Materials

The following supporting information can be downloaded at: https://www.mdpi.com/article/10.3390/jcm13071958/s1, Table S1: Overview of descriptive statistics of qMRI metrics fat fraction (FF), water T2 relaxation time (T2), fractional anisotropy (FA), and mean diffusivity (MD) for each muscle. Table S2: Overview of Spearman rank correlation coefficients for qMRI parameters fat fraction (FF), water T2 relaxation time (T2), fractional anisotropy (FA), and mean diffusivity (MD), and patient questionnaires Neuromuscular Symptom Score (NSS), and ACTIVLIM.

## References

[1] Greenberg S.A. (2019). Inclusion body myositis: Clinical features and pathogenesis. Nat. Rev. Rheumatol..

[2] Hightower R.M., Alexander M.S. (2018). Genetic modifiers of Duchenne and facioscapulohumeral muscular dystrophies. Muscle Nerve.

[3] Angelini C., Burlina A., Blau N., Ferreira C.R. (2022). Clinical and biochemical footprints of inherited metabolic disorders: X. Metabolic myopathies. Mol. Genet. Metab..

[4] Pasnoor M., Barohn R.J., Dimachkie M.M. (2014). Toxic Myopathies. Neurol. Clin..

[5] Jacob S., Kapadia R., Soule T., Luo H., Schellenberg K.L., Douville R.N., Pfeffer G. (2022). Neuromuscular Complications of SARS-CoV-2 and Other Viral Infections. Front. Neurol..

[6] Badenoch J.B., Rengasamy E.R., Watson C., Jansen K., Chakraborty S., Sundaram R.D., Hafeez D., Burchill E., Saini A., Thomas L. (2022). Persistent neuropsychiatric symptoms after COVID-19: A systematic review and meta-analysis. Brain Commun..

[7] Soriano J.B., Murthy S., Marshall J.C., Relan P., Diaz J.V. (2021). A clinical case definition of post-COVID-19 condition by a Delphi consensus. Lancet Infect. Dis..

[8] Balcom E.F., Nath A., Power C. (2021). Acute and chronic neurological disorders in COVID-19: Potential mechanisms of disease. Brain.

[9] Mercuri E., Sumner C.J., Muntoni F., Darras B.T., Finkel R.S. (2022). Spinal muscular atrophy. Nat. Rev. Dis. Prim..

[10] Dahlqvist J.R., Widholm P., Leinhard O.D., Vissing J. (2020). MRI in Neuromuscular Diseases: An Emerging Diagnostic Tool and Biomarker for Prognosis and Efficacy. Ann. Neurol..

[11] Roy B., Lucchini M., Lilleker J.B., Goyal N.A., Naddaf E., Adler B., Alfano L.N., Malandraki G.A., Focht Garand K.L., Mochel D. (2023). Current status of clinical outcome measures in inclusion body myositis: A systematised review. Clin. Exp. Rheumatol..

[12] Barp A., Ferrero A., Casagrande S., Morini R., Zuccarino R. (2021). Circulating Biomarkers in Neuromuscular Disorders: What Is Known, What Is New. Biomolecules.

[13] Vincenten S.C.C., Mul K., van As D., Jansen J.J., Heskamp L., Heerschap A., van Engelen B.G.M., Voermans N.C. (2023). Five-year follow-up study on quantitative muscle magnetic resonance imaging in facioscapulohumeral muscular dystrophy: The link to clinical outcome. J. Cachexia. Sarcopenia Muscle.

[14] Marty B., Baudin P.-Y., Caldas de Almeida Araujo E., Fromes Y., Wahbi K., Reyngoudt H. (2023). Assessment of Extracellular Volume Fraction in Becker Muscular Dystrophy by Using MR Fingerprinting. Radiology.

[15] Forsting J., Rohm M., Froeling M., Güttsches A.K., Südkamp N., Roos A., Vorgerd M., Schlaffke L., Rehmann R. (2022). Quantitative muscle MRI captures early muscle degeneration in calpainopathy. Sci. Rep..

[16] De Wel B., Huysmans L., Peeters R., Goosens V., Ghysels S., Byloos K., Putzeys G., D’Hondt A., De Bleecker J.L., Dupont P. (2022). Prospective Natural History Study in 24 Adult Patients with LGMDR12 over 2 Years of Follow-up: Quantitative MRI and Clinical Outcome Measures. Neurology.

[17] Alonso-Jiménez A., Nuñez-Peralta C., Montesinos P., Alonso-Pérez J., García C., Montiel E., Belmonte I., Pedrosa I., Segovia S., Llauger J. (2021). Different Approaches to Analyze Muscle Fat Replacement with Dixon MRI in Pompe Disease. Front. Neurol..

[18] Burakiewicz J., Sinclair C.D.J., Fischer D., Walter G.A., Kan H.E., Hollingsworth K.G. (2017). Quantifying fat replacement of muscle by quantitative MRI in muscular dystrophy. J. Neurol..

[19] Veeger T.T.J., van de Velde N.M., Keene K.R., Niks E.H., Hooijmans M.T., Webb A.G., de Groot J.H., Kan H.E. (2022). Baseline fat fraction is a strong predictor of disease progression in Becker muscular dystrophy. NMR Biomed..

[20] Wang F., Fang S., Li J., Yuan L., Hou B., Zhu J., Jiao Y., Liu Z., Qian M., Santini F. (2023). Correlation analysis of quantitative MRI measurements of thigh muscles with histopathology in patients with idiopathic inflammatory myopathy. Eur. Radiol. Exp..

[21] Schlaeger S., Weidlich D., Zoffl A., Becherucci E.A., Kottmaier E., Montagnese F., Deschauer M., Schoser B., Zimmer C., Baum T. (2022). Beyond mean value analysis—A voxel-based analysis of the quantitative MR biomarker water T_2_ in the presence of fatty infiltration in skeletal muscle tissue of patients with neuromuscular diseases. NMR Biomed..

[22] Martín-Noguerol T., Barousse R., Wessell D.E., Rossi I., Luna A. (2023). Clinical applications of skeletal muscle diffusion tensor imaging. Skeletal Radiol..

[23] Cameron D., Reiter D.A., Adelnia F., Ubaida-Mohien C., Bergeron C.M., Choi S., Fishbein K.W., Spencer R.G., Ferrucci L. (2023). Age-related changes in human skeletal muscle microstructure and architecture assessed by diffusion-tensor magnetic resonance imaging and their association with muscle strength. Aging Cell.

[24] Cameron D., Abbassi-Daloii T., Heezen L.G.M., van de Velde N.M., Koeks Z., Veeger T.T.J., Hooijmans M.T., el Abdellaoui S., van Duinen S.G., Verschuuren J.J.G.M. (2023). Diffusion-tensor magnetic resonance imaging captures increased skeletal muscle fibre diameters in Becker muscular dystrophy. J. Cachexia. Sarcopenia Muscle.

[25] Hooijmans M.T., Damon B.M., Froeling M., Versluis M.J., Burakiewicz J., Verschuuren J.J.G.M., Niks E.H., Webb A.G., Kan H.E. (2015). Evaluation of skeletal muscle DTI in patients with duchenne muscular dystrophy. NMR Biomed..

[26] Aeles J., Bolsterlee B., Kelp N.Y., Dick T.J.M., Hug F. (2022). Regional variation in lateral and medial gastrocnemius muscle fibre lengths obtained from diffusion tensor imaging. J. Anat..

[27] Bolsterlee B., Souza A.D., Herbert R.D. (2019). Reliability and robustness of muscle architecture measurements obtained using diffusion tensor imaging with anatomically constrained tractography. J. Biomech..

[28] Chianca V., Vincenzo B., Cuocolo R., Zappia M., Guarino S., Di Pietto F., Del Grande F. (2023). MRI Quantitative Evaluation of Muscle Fatty Infiltration. Magnetochemistry.

[29] Schlaffke L., Rehmann R., Rohm M., Otto L.A.M., De Luca A., Burakiewicz J., Baligand C., Monte J., den Harder C., Hooijmans M.T. (2019). Multi-center evaluation of stability and reproducibility of quantitative MRI measures in healthy calf muscles. NMR Biomed..

[30] Van Capelle C.I., Van Der Beek N.A.M.E., De Vries J.M., Van Doorn P.A., Duivenvoorden H.J., Leshner R.T., Hagemans M.L.C., Van Der Ploeg A.T. (2012). The quick motor function test: A new tool to rate clinical severity and motor function in Pompe patients. J. Inherit. Metab. Dis..

[31] Froeling M. (2019). QMRTools: A Mathematica toolbox for quantitative MRI analysis. J. Open Source Softw..

[32] Reeder S.B., Pineda A.R., Wen Z., Shimakawa A., Yu H., Brittain J.H., Gold G.E., Beaulieu C.H., Pelc N.J. (2005). Iterative Decomposition of Water and Fat With Echo Asymmetry and Least-Squares Estimation (IDEAL): Application with Fast Spin-Echo Imaging. Magn. Reson. Med..

[33] Marty B., Baudin P., Reyngoudt H., Azzabou N., Araujo E.C.A., Carlier P.G., de Sousa P.L. (2016). Simultaneous muscle water *T*_2_ and fat fraction mapping using transverse relaxometry with stimulated echo compensation. NMR Biomed..

[34] Leemans A., Jones D.K. (2009). The B-matrix must be rotated when correcting for subject motion in DTI data. Magn. Reson. Med..

[35] Veraart J., Novikov D.S., Christiaens D., Ades-Aron B., Sijbers J., Fieremans E. (2016). Denoising of diffusion MRI using random matrix theory. Neuroimage.

[36] Veraart J., Sijbers J., Sunaert S., Leemans A., Jeurissen B. (2013). Weighted linear least squares estimation of diffusion MRI parameters: Strengths, limitations, and pitfalls. Neuroimage.

[37] Rohm M., Markmann M., Forsting J., Rehmann R., Froeling M., Schlaffke L. (2021). 3D Automated Segmentation of Lower Leg Muscles Using Machine Learning on a Heterogeneous Dataset. Diagnostics.

[38] Klein S., Staring M., Murphy K., Viergever M.A., Pluim J. (2010). *elastix*: A Toolbox for Intensity-Based Medical Image Registration. IEEE Trans. Med. Imaging.

[39] Froeling M., Tax C.M.W., Vos S.B., Luijten P.R., Leemans A. (2017). “MASSIVE” brain dataset: Multiple acquisitions for standardization of structural imaging validation and evaluation. Magn. Reson. Med..

[40] Forsting J., Rehmann R., Froeling M., Vorgerd M., Tegenthoff M., Schlaffke L. (2020). Diffusion tensor imaging of the human thigh: Consideration of DTI-based fiber tracking stop criteria. Magn. Reson. Mater. Phys. Biol. Med..

[41] Barp A., Laforet P., Bello L., Tasca G., Vissing J., Monforte M., Ricci E., Choumert A., Stojkovic T., Malfatti E. (2020). European muscle MRI study in limb girdle muscular dystrophy type R1/2A (LGMDR1/LGMD2A). J. Neurol..

[42] Morishima R., Schoser B. (2023). A Straightforward Approach to Analyze Skeletal Muscle MRI in Limb-Girdle Muscular Dystrophy for Differential Diagnosis: A Systematic Review. Muscles.

[43] Cox F.M., Reijnierse M., van Rijswijk C.S.P., Wintzen A.R., Verschuuren J.J., Badrising U.A. (2011). Magnetic resonance imaging of skeletal muscles in sporadic inclusion body myositis. Rheumatology.

[44] Ansari B., Salort-Campana E., Ogier A., Le Troter PhD A., De Sainte Marie B., Guye M., Delmont E., Grapperon A.M., Verschueren A., Bendahan D. (2020). Quantitative muscle MRI study of patients with sporadic inclusion body myositis. Muscle Nerve.

[45] Morrow J.M., Sinclair C.D.J., Fischmann A., Machado P.M., Reilly M.M., Yousry T.A., Thornton J.S., Hanna M.G. (2016). MRI biomarker assessment of neuromuscular disease progression: A prospective observational cohort study. Lancet Neurol..

[46] Figueroa-Bonaparte S., Llauger J., Segovia S., Belmonte I., Pedrosa I., Montiel E., Montesinos P., Sánchez-González J., Alonso-Jiménez A., Gallardo E. (2018). Quantitative muscle MRI to follow up late onset Pompe patients: A prospective study. Sci. Rep..

[47] Rehmann R., Froeling M., Rohm M., Forsting J., Kley R.A., Schmidt-Wilcke T., Karabul N., Tegenthoff M., Vorgerd M., Schlaffke L. (2020). Muscle Diffusion tensor imaging reveals changes in non-fat infiltrated muscles in late-onset Pompe disease. Muscle Nerve.

[48] Reyngoudt H., Smith F.E., Caldas de Almeida Araújo E., Wilson I., Fernández-Torrón R., James M.K., Moore U.R., Díaz-Manera J., Marty B., Azzabou N. (2022). Three-year quantitative magnetic resonance imaging and phosphorus magnetic resonance spectroscopy study in lower limb muscle in dysferlinopathy. J. Cachexia. Sarcopenia Muscle.

[49] Schlaeger S., Weidlich D., Klupp E., Montagnese F., Deschauer M., Schoser B., Bublitz S., Ruschke S., Zimmer C., Rummeny E.J. (2019). Decreased water T2 in fatty infiltrated skeletal muscles of patients with neuromuscular diseases. NMR Biomed..

[50] Carlier P.G., Marty B., Scheidegger O., De Sousa P.L., Baudin P.Y., Snezhko E., Vlodavets D. (2016). Skeletal Muscle Quantitative Nuclear Magnetic Resonance Imaging and Spectroscopy as an Outcome Measure for Clinical Trials. J. Neuromuscul. Dis..

[51] Locher N., Wagner B., Balsiger F., Scheidegger O. (2022). Quantitative water T2 relaxometry in the early detection of neuromuscular diseases: A retrospective biopsy-controlled analysis. Eur. Radiol..

[52] Enax-Krumova E., Forsting J., Rohm M., Schwenkreis P., Tegenthoff M., Meyer-Frießem C.H., Schlaffke L. (2023). Quantitative muscle magnetic resonance imaging depicts microstructural abnormalities but no signs of inflammation or dystrophy in post-COVID-19 condition. Eur. J. Neurol..

[53] Carlier P.G., Azzabou N., De Sousa P.L., Hicks A., Boisserie J.-M., Amadon A., Carlier R.-Y., Wary C., Orlikowski D., Laforêt P. (2015). Skeletal muscle quantitative nuclear magnetic resonance imaging follow-up of adult Pompe patients. J. Inherit. Metab. Dis..

[54] Benveniste O., Stenzel W., Hilton-Jones D., Sandri M., Boyer O., van Engelen B.G.M. (2015). Amyloid deposits and inflammatory infiltrates in sporadic inclusion body myositis: The inflammatory egg comes before the degenerative chicken. Acta Neuropathol..

[55] Otto L.A.M., van der Pol W.L., Schlaffke L., Wijngaarde C.A., Stam M., Wadman R.I., Cuppen I., van Eijk R.P.A., Asselman F.-L., Bartels B. (2020). Quantitative MRI of skeletal muscle in a cross-sectional cohort of patients with spinal muscular atrophy types 2 and 3. NMR Biomed..

[56] Williams S.E., Heemskerk A.M., Welch E.B., Li K., Damon B.M., Park J.H. (2013). Quantitative effects of inclusion of fat on muscle diffusion tensor MRI measurements. J. Magn. Reson. Imaging.

[57] Berry D.B., Regner B., Galinsky V., Ward S.R., Frank L.R. (2018). Relationships Between Tissue Microstructure and the Diffusion Tensor in Simulated Skeletal Muscle. Magn. Reson. Med..

[58] Rohm M., Russo G., Helluy X., Froeling M., Umathum V., Südkamp N., Manahan-Vaughan D., Rehmann R., Forsting J., Jacobsen F. (2023). Muscle diffusion MRI correlates with autophagic buildup in a Pompe disease mouse model. Sci. Rep..

[59] Sherlock S.P., Palmer J., Wagner K.R., Abdel-Hamid H.Z., Bertini E., Tian C., Mah J.K., Kostera-Pruszczyk A., Muntoni F., Guglieri M. (2022). Quantitative magnetic resonance imaging measures as biomarkers of disease progression in boys with Duchenne muscular dystrophy: A phase 2 trial of domagrozumab. J. Neurol..

[60] Suslov V.M., Lieberman L.N., Carlier P.G., Ponomarenko G.N., Ivanov D.O., Rudenko D.I., Suslova G.A., Adulas E.I. (2023). Efficacy and safety of hydrokinesitherapy in patients with dystrophinopathy. Front. Neurol..

